# A parathyroid-hormone-related-protein (PTH-rP)-specific cytotoxic T cell response induced by in vitro stimulation of tumour-infiltrating lymphocytes derived from prostate cancer metastases, with epitope peptide-loaded autologous dendritic cells and low-dose IL-2

**DOI:** 10.1054/bjoc.2001.2136

**Published:** 2001-11

**Authors:** P Correale, L Micheli, M T Del Vecchio, M Sabatino, R Petrioli, D Pozzessere, S Marsili, G Giorgi, L Lozzi, P Neri, G Francini

**Affiliations:** Division of Medical Oncology, Department of Pharmacology, ‘Giorgio Segre’; Institute of Pathologic Anatomy and Histology, Department of Molecular Biology, Faculty of Medicine, University of Siena, Siena, 53100, Italy

**Keywords:** prostate carcinoma, TIL, CTL epitope peptides, PTH-rP, human cancer immunotherapy

## Abstract

Bone metastases are one of the most common events in patients with prostate carcinoma. PTH-rP, a protein produced by prostate carcinoma and other epithelial cancers, is a key agent for the development of bone metastases. A PTH-rP-derived peptide, designated PTR-4 was identified, which is capable to bind HLA-A2.1 molecules and to generate PTH-rP-specific cytotoxic T cell (CTL) lines from healthy HLA-A2.1^+^ individual peripheral-blood-mononuclear-cells (PBMC). In this model, we investigated the in vitro possibility of generating an efficient PTH-rP specific CTL response by cyclical stimulations with IL-2 and PTR-4 peptide-pulsed autologous dendritic cells (DC), of HLA-A2.1^+^ tumour infiltrating lymphocytes (TIL) derived from a patient with metastatic prostate carcinoma. A T cell line generated in this way (called TM-PTR-4) had a CD3^+^, CD5^+^, CD4^−^, CD8^+^, CD45^Ro+^, CD56^−^ immunophenotype and a HLA-A2.1 restricted cytotoxic activity to PTR-4-peptide pulsed CIR-A2 (HLA-A2.1^+^) target cells, PTH-rP^+^/HLA-A2.1^+^ CIR-A2 transfected with PTH-rP gene, prostate carcinoma LNCaP cells, and autologous metastatic prostate cancer cells (M-CaP). These lymphocytes were not cytotoxic to HLA-A2.1^+^ targets not producing PTH-rP, such as peptide-unpulsed CIR-A2 and colon carcinoma SW-1463, cell lines. Our results provide evidence that PTR-4 peptide-pulsed autologous DC may break the tolerance of human TIL against the autologous tumour by inducing a PTH-rP-specific CTL immune reaction. In conclusion PTR-4 peptide-pulsed autologous DC may be a promising approach for vaccine-therapy and antigen-specific CTL adoptive immunotherapy of hormone-resistant prostrate cancer. © 2001 Cancer Research Campaign http://www.bjcancer.com


British Journal of Cancer (2001) 85(11), 1722–1730
© 2001 Cancer Research Campaign
doi: 10.1054/ bjoc.2001.2136, available online at http://www.idealibrary.com on IDEAL

http://www.bjcancer.com

### A parathyroid-hormone-related-protein (PTH-rP)-specific cytotoxic T cell response induced by in vitro stimulation of tumour-infiltrating lymphocytes derived from prostate cancer metastases, with epitope peptide-loaded autologous dendritic cells and low-dose IL-2

**P Correale[1], L Micheli[2], MT Del Vecchio[3], M Sabatino[1], R Petrioli[1], D Pozzessere[1], S Marsili[1], G Giorgi[2], L Lozzi[4], P Neri[4] and G Francini[1]**

[1]Division of Medical Oncology; [2]Department of Pharmacology, 'Giorgio Segre'; [3]Institute of Pathologic Anatomy and Histology; [4]Department of Molecular Biology, Faculty of Medicine, University of Siena, 53100 Siena, Italy

**Summary** Bone metastases are one of the most common events in patients with prostate carcinoma. PTH-rP, a protein produced by prostate carcinoma and other epithelial cancers, is a key agent for the development of bone metastases. A PTH-rP-derived peptide, designated PTR-4 was identified, which is capable to bind HLA-A2.1 molecules and to generate PTH-rP-specific cytotoxic T cell (CTL) lines from healthy HLA-A2.1$^+$ individual peripheral-blood-mononuclear-cells (PBMC). In this model, we investigated the in vitro possibility of generating an efficient PTH-rP specific CTL response by cyclical stimulations with IL-2 and PTR-4 peptide-pulsed autologous dendritic cells (DC), of HLA-A2.1$^+$ tumour infiltrating lymphocytes (TIL) derived from a patient with metastatic prostate carcinoma. A T cell line generated in this way (called TM-PTR-4) had a CD3$^+$, CD5$^+$, CD4$^-$, CD8$^+$, CD45$^{Ro+}$, CD56$^-$ immunophenotype and a HLA-A2.1 restricted cytotoxic activity to PTR-4-peptide pulsed CIR-A2 (HLA-A2.1$^+$) target cells, PTH-rP$^+$/HLA-A2.1$^+$ CIR-A2 transfected with PTH-rP gene, prostate carcinoma LNCaP cells, and autologous metastatic prostate cancer cells (M-CaP). These lymphocytes were not cytotoxic to HLA-A2.1$^+$ targets not producing PTH-rP, such as peptide-unpulsed CIR-A2 and colon carcinoma SW-1463, cell lines. Our results provide evidence that PTR-4 peptide-pulsed autologous DC may break the tolerance of human TIL against the autologous tumour by inducing a PTH-rP-specific CTL immune reaction. In conclusion PTR-4 peptide-pulsed autologous DC may be a promising approach for vaccine-therapy and antigen-specific CTL adoptive immunotherapy of hormone-resistant prostrate cancer. © 2001 Cancer Research Campaign http://www.bjcancer.com

**Keywords**: prostate carcinoma; TIL; CTL epitope peptides; PTH-rP; human cancer immunotherapy

Prostate cancer is the most common cancer in elder men (Coffey, 1993); the majority of whom with advanced disease develop bone metastases if they live long enough (Hortobagyi, 1991; Vinholes et al, 1996; Rubens, 1998).

Once bone metastases become clinically evident, the disease becomes almost invariably refractory to hormones and only few palliative means are available to combat cancer progression and the occurrence of serious complications related to metastatic bone involvement (Hortobagyi, 1991; Francini et al, 1993; Rubens, 1998). Active specific immunotherapy and tumour-associated antigens (TAA)-specific adoptive immunotherapy are currently being investigated throughout the world as novel strategies for treating prostate cancer. In these cases it is generally accepted that a T lymphocyte response to TAA is indispensable for obtaining an efficient and prolonged tumour rejection in vivo (Marincola, 1994; Restifo and Sznol, 1997, pp 3023–3038). Tumour-infiltrating lymphocytes (TIL) are the final result of the long battle occurring between the host immune reaction and the continued cancer cell development and growth (Vose and Moore, 1985; Topalian et al, 1987; Topalian and Rosenberg, 1989; Whiteside, 1991; Rosenberg et al, 1994; Whiteside and Parmiani, 1994; Pierce et al, 1995), and the preclinical data concerning in vitro TIL activation against the autologous tumour cells collected over the last 10 years has encouraged a large number of clinical reinfusion trials using TIL plus interleukin 2 (IL-2) (Topalian et al, 1987). For many different reasons, these studies led to very conflicting results in terms of clinical effectiveness and reproducibility (Rosenberg et al, 1994; Pierce et al, 1995; Restifo and Sznol, 1997).

The greater knowledge regarding the human cell-mediated immune system acquired over the last few years has made it possible to improve the anti-tumour potential of these effector cells. It is now known that tumour cells can be recognized and killed by activated lymphocyte effectors but cannot start a demonstrable de novo CTL immune reaction (Melero et al, 1997), which requires professional antigen-presenting cells (APC) such as dendritic cells (DC), that are capable of uptaking, processing and presenting to the CTL precursors antigen proteins released by tumour cells (Grabbe et al, 1995; Romani et al, 1996; Bell et al, 1999). Furthermore, it has also been discovered that T lymphocytes recognize tumour as well as viral protein antigens as small peptides (peptide epitopes) which are products of intracellular degradation of antigen protein, bound to major histocompatibility complex (MHC) molecules on target cells (Melero et al, 1997).

*Received 30 May 2001*
*Revised 14 August 2001*
*Accepted 31 August 2001*

*Correspondence to:* G Francini

Epitope peptide binding to specific HLA isotypes is determined by definite *consensus motifs* present in the amino acid sequence of antigen peptides (Cerundolo et al, 1990).

A number of these sequences have been described for the most common human leukocyte antigen (HLA) isotypes, and CTL epitope peptides with HLA-A2.1 *binding motifs* capable of generating antigen-specific CTL lines with anti-tumour activity have been derived from most common TAA (Jung and Schluesener, 1991; Van der Bruggen et al, 1991; Fenton et al, 1993; Houbiers et al, 1993; Tsang et al, 1994, 1995; Abrams et al, 1995; Correale et al, 1997; Tjoa et al, 1998; Francini et al, 2001). Recent in vitro and in vivo findings also demonstrated the possibility to induce a CTL immune response against TAAs produced by prostate cancer, such as prostate-specific antigen (PSA) (Correale et al, 1997), parathyroid hormone-related protein (PTH-rP) (Francini et al, 2000), and prostate-specific membrane antigen (PSMA) (Tjoa et al, 1998). Various studies have evaluated the possibility of inducing a TAA-specific CTL response from peripheral blood mononuclear cells (PBMC), and identified a number of new TAA and peptide epitopes to be used in clinical trials of active specific immunotherapy. In this contest, we have recently identified and characterized 4 HLA-A2.1 binding epitope peptides from PTH-rP, a 177 amino acid protein that is produced by 90% or prostate carcinoma cells and is a key agent for the survival and growth of prostate cancer cells in the bone tissue (Guise, 1997).

These peptides were designated PTR-1, -2, -3 and -4, and were used to generate an HLA-A2.1 restricted, PTH-rP-specific CTL response leading to the generation from PBMC of healthy HLA-A2.1$^+$ individuals, of PTH-rP-specific CTL lines with antitumour activity in vitro (Francini et al, 2001).

Although CTL lines have been established for the most common prostate cancer-associated antigens very few studies have been conversely designed to investigate the possibility to induce an antigen peptide-specific CTL response in TIL derived from patients with non-immunogenic tumours such as prostate carcinoma; furthermore, no clear demonstration that an antigen-specific CTL response with antitumour activity may be generated from human TIL isolated from prostate cancer metastatic tissue has never been provided.

In a recent clinical trial we observed that the sequential administration of granulocyte-monocyte colony stimulating factor (GM-CSF) (150 μg day$^{-1}$ s.c. for 5 days) followed by very low dose s.c. human recombinant interleukin-2 (IL-2) in patients with very advanced cancers led to a significant increase in and activation of peripheral DC (Correale et al, 2001). Before being enrolled in the clinical trial, one of these patients with advanced prostate carcinoma underwent a diagnostic bone biopsy from which cancer cells and TIL could be isolated and cultured in vitro; PBMC isolated from this patient before, during and after cytokine administration were also isolated and made available for immunological studies.

Aim of the present in vitro study was to investigate the possibility of generating an efficient human CTL response to PTH-rP by stimulating TIL derived from prostate cancer bone metastases with PTH-rP peptide-pulsed autologous DC isolated after GM-CSF and IL-2 treatment. The rationale of this study was based on the hypothesis that TAA epitope peptide stimulation in the presence of activated autologous DC may restore the antigen-specific cytolytic activity of these TIL against the autologous tumour cells expressing the specific target antigen.

### MATERIAL AND METHODS

### Cell cultures

The prostate carcinoma LNCaP and the colon carcinoma SW1463 cell lines were purchased from American Type Culture Collection (Rockville, MD, USA). The cultures were mycoplasma free and were maintained in complete medium (Dulbecco's modified Eagle medium) (Life Technologies Inc (Gibco BRL), Grand Island, NY) supplemented with 10% heat inactivated fetal bovine serum (FBS), 2 mM glutamine, 100 U ml$^{-1}$ penicillin, and 100 μg ml$^{-1}$ streptomycin (Life Technologies Inc). The CIR-A2 cell line was provided by Dr Jeffry Schlom (Experimental Oncology Section, National Cancer Institute, National Institute of Health, Bethesda MD, USA) and maintained in Iscove's modified Dulbecco's complete medium (IMDM).

### Peptide synthesis

Synthesis of PTR-4 peptides (amino acid sequence: TSTTSLELD) was performed on a solid phase automatic peptide synthesizer (model syto, MultiSyntech, Witten, D) using the Fluorenylmethoxycarbonyl (Fmoc)/Diisopropylcarbodiimide (DIC)/1-Hydroxybenzotriazole (HOBT) strategy. Peptides were cleaved from the resins and defracted by treatment with trifluoroacetic acid containing ethandiethiol, water trisbuthyl silone and anisole (93/2.5/2/1.5/1). The crude peptides were purified by HPLC using a Vydac C18 column (25 cm × 1 cm, 10 μm). The products dissolved in bi-distilled water, sterile filtered and frozen at –70˚C at a concentration of 2 mg ml$^{-1}$. The purity of the peptides was more than 90% as analysed by high-performance liquid chromatography (HPLC). The CAP-1 control peptide was kindly donated by Dr J Schlom.

### TIL cell culture and expansion

Tissue obtained from biopsy samples of metastatic bone were mechanically fragmented and seeded in T25 (Corning, Costar Milano, Italia) dissolved in AIM-V medium supplemented with 5% pooled human heat-inactivated AB serum (Valley Biomedical, Winchester, VA), 2 mM glutamine, 100 U ml$^{-1}$ penicillin, and 100 μg ml$^{-1}$ of streptomycin (Flow, Irvine, UK), and containing 6000 IU ml$^{-1}$ recombinant IL-2 (Cetus Corp, Emeryville, CA, USA). The cells were incubated at 37˚C and 5% $CO_2$ for 96 hours. The mononucleate cells were subsequently, separated using density gradients. The cell suspension obtained was cultured at the concentration of $1 \times 10^6$ cell ml$^{-1}$ in 24-multiwell plates (Corning, Costar Milano, Italia) in the same IL-2-containing medium. One week later, the cells were evaluated for T cell immune phenotype and stimulated with peptide-pulsed autologous DC as described above for PTH-rP peptide-specific CTL lines.

### Generation of DC and T cell lines

PBMC were isolated before, during and after treatment with GM-CSF and IL-2 from heparinized blood taken from a HLA-A2$^+$ male donor affected by advanced prostate carcinoma using a lymphocyte separation medium gradient (Celbio Biotecnologie SRL, Milano, Italia) as previously described (Boyum et al, 1968).

PBMC for DC enrichment ($10^7$ cell ml$^{-1}$) were seeded in T75 flasks in a 10 ml volume of complete medium (RPMI-1640) with 10% heat inactivated FBS. After 4 hours' incubation at 37˚C in 5%

*© 2001 Cancer Research Campaign*

$CO_2$, the non-adherent cells were removed and adherent cells were maintained for 7 days in complete medium (RPMI-1640 with 10% heat inactivated FBS, 2 mM glutamine, 100 U $ml^{-1}$ penicillin, and 100 μg $ml^{-1}$ streptomycin) containing 50 ng $ml^{-1}$ of GM-CSF (Molgramostim, kindly furnished by the Shering Plough, SPA, Milano, Italia) and 0.5 ng $ml^{-1}$ of IL-4 (purchased from the R and D systems, Minneapolis, MN, USA). The medium containing GM-CSF and IL-4 was replaced every 48 hours. After 7 days of culture the DC phenotype cells was evaluated by means of direct immunofluorescence flow cytometry. In order to estimate the DC enrichment before CTL stimulation, the cultures were examined for the expression of CD1a, HLA-I, HLA-DR, CD11c, CD80, CD83 and CD86, which are markers known to be highly expressed by DC and involved in T cell costimulation and activation (Grabbe et al, 1995; Romani et al, 1995; Bell et al, 1999).

The TIL for CTL primary cultures, were washed and then resuspended in AIM-V medium (Life Technologies, SRL, Milano, Italia) supplemented with 5% pooled human heat-inactivated AB serum (Valley Biomedical, Winchester, VA), 2 mM glutamine, 100 U $ml^{-1}$ penicillin, and 100 μg $m^{-1}$ of streptomycin (Gibco). The cells ($2 \times 10^5$) in a volume of 100 μl of complete medium were added to each well of a 96-well flat-bottom assay plate (Corning, Costar Milano, Italia).

Irradiated autologous DC were pulsed with PTR-4 peptides (25 μg $ml^{-1}$) and added to the lymphocyte cultures at the final ratio of 1:5. DC cultures were irradiated prior TIL or PBMC in vitro stimulation, in order to prevent the possibility that contaminating NK or T cells could proliferate in presence of TIL and cytokines, affecting the clonal expansion of PTH-rP peptide-specific T cell clones.

Cultures were incubated for 5 days at 37˚C in a humidified atmosphere containing 5% $CO_2$, and then provided with human recombinant IL-2 (20 U $ml^{-1}$) for 11 days, with the IL-2-containing medium being replenished every 3 days. The incubation time of 5 days with peptide-pulsed DC plus 10 days with IL-2 constitutes one in vitro stimulation cycle (IVS). The T cell cultures were re-stimulated with PTH-rP peptide (25 μg $ml^{-1}$) pulsed autologous DC on day 16. Irradiated (5000 rads) autologous DC were added in a volume of 100 μl of complete medium (AIM-V) and used as antigen-presenting cells.

The progenitor TIL cultures were maintained in AIM-V medium supplemented with 5% pooled human heat inactivated AB serum, 2 mM glutamine, 100 U $ml^{-1}$ penicillin, and 100 μg $ml^{-1}$ of streptomycin, and containing 20 IU $ml^{-1}$ of IL-2. The TIL cultures were restimulated every 15 days with autologous irradiated DC cells plus autologous irradiated prostate cancer cells in a 5:1 ratio.

### *T cell lines*

PBMC, for CTL primary cultures, were suspended in AIM-V medium (Life Technologies Inc) supplemented with 5% pooled human heat inactivated AB serum (Valley Biomedical, Winchester, VA), 2 mM L-glutamine, 100 U $ml^{-1}$ penicillin/streptomycin (Gibco). $2 \times 10^5$ cells, in a volume of 100 μl, were seeded into each well of a 96-well microplate (Corning, Costar Corp, Cambridge, MA).

Autologous DC were first irradiated (5000 R) and pulsed with 25 μg $ml^{-1}$ of PTR-4 and then added to the lymphocyte cultures to the final ratio of 1:5. One in vitro stimulation cycle (IVS) was represented by a 5-day period of cell incubation with antigen-loaded DC plus a 10-day period of cell stimulation with 50 IU of IL-2 (Cetus corp). Fresh complete medium containing cytokines was replaced every 48 hours. On the 16th day, T cell cultures were re-stimulated with autologous irradiated DC pulsed with PTR-4 used as-antigen presenting cells.

### Culture and expansion of prostate cancer cell derived from bone metastases

The tissue obtained from metastatic bone samples, taken by bone biopsy, were mechanically fragmented and seeded in T25 flasks (Corning, Costar Milano, Italia) in RPMI medium supplemented with 5% pooled human heat inactivated AB serum, 2 mM glutamine, 100 U $ml^{-1}$ penicillin, and 100 μg $ml^{-1}$ of streptomycin, and containing 100 IU of human recombinant Insulin (Sigma-Aldrich, SRL, Milano, Italia) and $10^{-8}$ M androstenedyone (Sigma-Aldrich, SRL, Milano, Italia). The cells were incubated at 37˚C and 5% $CO_2$ for 6 weeks with the medium being replenished every week. Adherent cells with epithelial cell features appeared evident after 2 weeks of culture. After 6 weeks cells were studied by a pathologist and immunohistochemically analysed for the expression of cytokeratines and PSA.

### Immunohistochemistry

The slides containing the cultured cells, were fixed in cold acetone for 10 minutes, and were incubated with anti-human PSA mouse monoclonal antibody (Immunotech, Marseille, France) diluted 1:500 in TBS; then the reaction was revealed using streptovidin–biotin complex. Slides were weakly counterstained with Harris' haematoxilin, mounted with aqueous mounting medium and examined under a light microscope. Negative controls were obtained for each case by replacing the specific antibody with non-immune serum immunoglobulin at the same concentration as the primary antibody. Immune reactivity was assessed using routine light microscopy.

### *Target cell transfection with PTH-rP plasmid*

The GC90 plasmid engineered to express PTH-rP has been previously described by our group (Correale et al, 2001b). The pcDNA3 expression vector (In Vitrogen) was utilised to obtain the recombinant plasmid GC90. Plasmid DNA was purified by Qiagen Endo Free plasmid kit (QIAGEN) as described by the manufacturer.

Approximately $10^5$ CIR-A2 target cells were grown in 6-well microplates at 37˚C transfected with 1 μg of plasmid DNA using the Effectene Transfection reagent (QIAGEN) as described by the manufacturer. After 2 days, PTH-rP antigen expression was analysed by evaluating the presence of the specific mRNA by RT-PCR and by immunofluorescence. Briefly, the cells were washed twice with PBS, fixed with cold methanol/acetone and treated with a rabbit anti-PTH-rP serum (Calbiochem) followed by FITC-conjugated goat-anti-rabbit IgG (1/100) (DBA). The coverslips were mounted on slides and examined using a Diaplan microscope (Leitz).

### Cytotoxic assays

Various target cells were labelled with 100 μCi of $Na_2Cr^{51}O_4$ (Amersham, Aylesbury, UK) for 60 minutes at room temperature. Target cells ($0.5 \times 10^4$) in 100 μl of complete medium (see below) were added to each of 96 wells in T-flat bottom assay plates (Corning Costar Italia, Milano). The labelled targets were incubated at 37˚C in 5% $CO_2$ before adding effector cells. The T cells were then suspended in 100 μl of AIM-V medium and added to the target cells. The plates were incubated at 37˚C for 18 hours. The supernatants were harvested for γ-counting using harvester frames

 *© 2001 Cancer Research Campaign*

(Skatron, Inc, Sterling, VA, USA). The determinations were carried out in triplicate and the standard deviations were calculated. All of the experiments were repeated at least 3 times.

Specific lysis was calculated as follows:

$$\% \text{ specific lysis} = \frac{\text{observed release (cpm)} - \text{spontaneous release (cpm)}}{\text{total release (cpm)} - \text{spontaneous release (cpm)}} \times 100$$

Spontaneous release was determined from wells to which 100 µl of complete medium had been added instead of effector cells; total releasable radioactivity was measured after treating the targets with 2.5% Triton X-100.

### *Blocking experiments*

For HLA blocking experiments, UPC-10 control mAbs (Cappel/Organon Technique Corp, West Chester, PA, USA) or anti-HLA-A2 mAbs (A2.69, #189HA-1; One Lambda Inc, Canoga Park, CA, USA) at the dilution of 1:100 dilution were added to the labelled target cells (M-CaP and LNCaP cells) and incubated for 1 hour before the cytotoxic assay.

### Flow cytometry

### *Single colour flow cytometric analysis*

The procedure for single-colour flow cytometric analysis has been previously described (Guadagni et al, 1990). Briefly $1 \times 10^6$ cells were washed 3 times with cold $Ca^{2+}$ and $Mg^{2+}$-free Dulbecco's phosphate-buffered saline (DPBS) and then stained for 1 h with 1 µg of mAb against CD3, CD4, CD8, CD56, CD19, CD11c, CD1a, CD83, CD86, CD80, CD54, CD58 (all purchased from Becton Dickinson, San Jose, CA), HLA class I (W6/32) (Scra, Sussex, England), and MOPC-21 (Cappel/Organon Tecknica Corp. West Chester, PA) in a volume of 100 µl of PBS containing 1% bovine serum albumin. The cells were then washed 3 times with cold DPBS and incubated for an additional hour in the presence of 1:100 dilution (volume of 100 µl PBS containing 1% bovine serum albumin) of fluoresceine-conjugated goat anti-mouse immunoglobulin (Ig) (Kirkengard and Perry Labs, Gaithersburg, MD, USA). The cells were again washed 3 times with DPBS and resuspended in DPBS at the concentration of $1 \times 10^6$ cells $ml^{-1}$, and immediately analysed using a Becton Dickinson FACScan equipped with a blue laser with an excitation of 15 nW at 488 nm. The data were gathered from 10 000 live cells and used to evaluate results.

### *Dual colour flow cytometric analysis*

This procedure closely resembled that used for the single-colour flow cytometry with the following exceptions. The mAbs used were anti-CD3 and anti-CD4 fluoresceine conjugate and anti-CD56 and anti-CD8 phycoerythrin conjugate, anti-IgG1 fluorescein conjugate and anti-IgG3 phycoerythrin (all purchased from Becton Dickinson, San Jose, CA). The cells were simultaneously stained for 1 hour and then washed 3 times, resuspended as above, and immediately analysed using a Becton Dickinson FACScan equipped, with a blue laser with an excitation of 15 nW at 488 nm, and the Lysis II program.

### HLA typing

The HLA phenotyping of the donor PBMC was performed by the Blood Bank of the Azienda Ospedaliera Senese, Policlinico 'Le Scotte', Siena, Italy using a standard antibody-dependent micro-cytotoxicity assay and a defined panel of anti-HLA antisera for HLA class I determinations.

### Statistical consideration

The differences between means were statistically analysed using the 2-tailed Student's *t*-test for paired samples and Stat View statistical software (Abacus Concepts, Berkeley, CA) and the results were expressed as the mean of 4 determinations derived from 3 different experiments +/– standard deviation. A $P$ value of $< 0.05$ was considered statistically significant.

### RESULTS

### PTH-rP specific TIL

A PTR-4 peptide specific CTL line, designated TM-PTR-4, was generated by the in vitro stimulation of human TIL with PTR-4 peptide-pulsed autologous DC and IL-2. TIL had been isolated from a metastatic sample taken by a bone biopsy, performed on a patient with advanced prostate carcinoma enrolled in a clinical trial of immunotherapy GM-CSF and IL-2 before he received any immunological treatment. DC were instead, isolated from the same patient after he had received at least a cycle of treatment with GM-CSF and IL-2. Immunofluorescence FACS analysis showed that this patient was HLA-A2.1$^+$, and that the administration of GM-CSF and IL-2 had indeed increased in his PBMC, the percentage of cells showing the immunophenotype and the function of activated bone marrow-derived DC (data from a clinical trial) (Correale et al, 2001). In order to increase and purify the DC population to use as APC, patient PBMC, isolated after he had received the cytokine immunological treatment, were cultured in medium containing GM-CSF and IL-4 as described above, and finally used to stimulate the autologous TIL.

After 4 IVS (8 weeks) this TM-PTR-4 lymphocyte culture was investigated for immunophenotype expression and cytotoxic activity.

Progenitor TIL (TM-TIL) were also cultured in presence of IL-2 and cyclically stimulated with the same irradiated autologous DC co-cultured in presence of autologous irradiated tumour cells. After 8 weeks of culture, also these lymphocytes were investigated for immunophenotype expression and cytotoxic activity. The immunophenotype study revealed that TM-PTR-4 culture mainly consisted of CD3$^+$, CD56$^-$, CD5$^+$ (22%), CD45RO$^+$ (45%) cells, which were prevalently CD4$^-$/CD8$^+$ (65.8%). The TM-TIL had a similar phenotype with a higher percentage of CD56$^+$ cells (20%), no CD5$^+$ (2.2%) expression and a much lower percentage of CD45$^{Ro+}$ (21%) memory T cells (Table 1). A similar phenotype was also found in the T cell line, generated by stimulating the same TIL with peptide-pulsed DC derived from PBMC isolated before patient treatment with GM-CSF and IL-2 (data not shown). In this patient no PTH-rP peptide-specific T cell line could be generated by using PTR-4 pulsed DC to stimulate PBMC isolated prior cytokine therapy (data not shown).

As previously described, a CTL line specific for the PTH-rP peptide was instead generated from PBMC isolated from a HLA-A2.1$^+$ healthy donor, by means of cyclic stimulation with low-dose IL-2 and PTR-4 peptide-pulsed autologous DC. DC used as CTL antigen-presenting cells were generated as described in the Method section. The CTL line generated in this way, was investigated for immunophenotype expression and cytolytic activity after 4 and 6 IVS (2 and 3 months of culture). Direct double-stain flow cytometry immunofluorescence showed that the CTL lines expressed a CD3$^+$ (98%) phenotype with different percentages of CD56$^+$ (25%), CD4$^+$/CD8$^-$ (41.2%), CD4$^+$/CD8$^+$ (15.2%) and CD4$^-$/CD8$^+$ (37.3%) cells (Table 1).

© 2001 Cancer Research Campaign

**Table 1** Flow cytometric analysis of surface markers on PTH-rP peptide specific CTL lines

| T cell line | Surface markers | | | | | | |
|---|---|---|---|---|---|---|---|
| | CD3⁺ | CD4⁺/CD8⁻ | CD4⁻/CD8⁺ | CD4⁺/CD8⁺ | CD56⁺ | CD5⁺ | CD45RO⁺ |
| TM-TIL | 87.2 | 25.3 | 69.3 | 4.5 | 36 | 2.2 | 21 |
| TM-PTR-4 | 96.46 | 28.57 | 65.81 | 2.41 | 3.29 | 22 | 45 |
| [a]ND-PTR-4 | 98% | 41.2% | 37.3% | 15.2% | 25% | – | 40% |

Results are expressed as percentage of each cell sample reactive with mAb. Routinely 2–4% cells are stained when treated either with no priming mAb or an isotype related control mAb. [a]ND-PTR-4 represents the CTL line derived from the PBMC of a HLA-A2.1⁺ healthy donor.

### CTL line cytotoxic activity

6 hour cytotoxic assays be means of $^{51}Cr$ release technique revealed that TM-PTR-4 lymphocytes were able to kill CIR-A2 target cells pulsed with PTR-4 peptide or transfected with GC90, a plasmid engineered to express PTH-rP gene. They also killed the HLA-A2.1⁺/PTH-rP⁺ prostate carcinoma LNCaP cell line (Figure 1). No CTL mediated lysis was conversely observed against unpulsed CIR-A2 cells or the cells pulsed with HLA-A2.1 molecule-binding peptides other than PTR-4, or CIR-A2 transfected with the plasmid vector (pcDNA3), or against colon cancer SW-1463 cells, which are HLA-A2.1⁺ and unable to produce PTH-rP (Figure 1). Target cell production of PTH-rP and membrane expression of HLA-A2.1 molecules is shown in Table 2.

6 hour cytotoxic assays also revealed TM-PTR-4 T-lymphocyte ability to kill autologous (M-CaP) prostate cancer cells (Figure 1). Autologous M-CaP prostate cancer cells derived from the same sample as that from which lymphocytes were isolated, were cultured in presence of androgens and insulin for 6 weeks before being used as targets in CTL assays. The cells used as CTL targets were fibroblast free and had an exponential growth rate (data not shown). M-CaP cells were considered prostate cancer cells after a morphological and immunohistochemical study had been performed by a pathologist. PSA expression in the epithelial aggregates was, in fact, demonstrated by immunohistochemistry as an intense red staining of the cytoplasm that masks nuclei and cell borders (Figure 2). The M-CaP target cells were also positive for the expression of HLA-A2.1 (60%), and PTH-rP (25 ng $ml^{-1} \times 10^6$ cells) (Table 2).

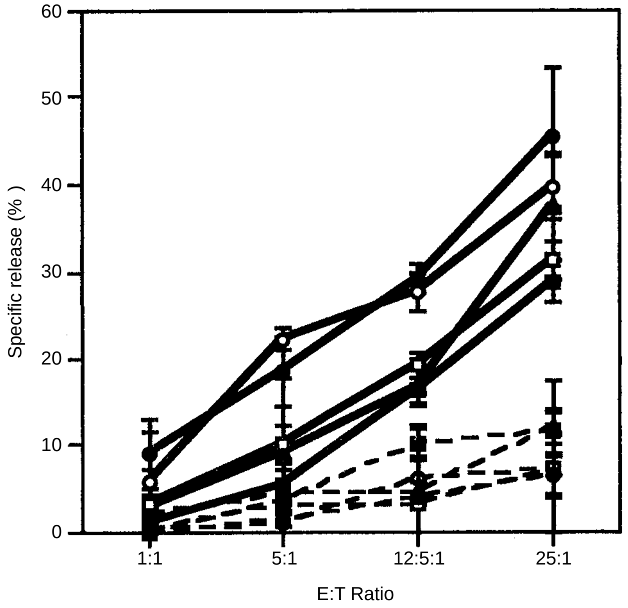

**Figure 1** 6 hour $^{51}Cr$ release cytotoxic assay. Cytotoxic activity of human TIL stimulated in vitro with IL-2 and PTH-rP peptide pulsed autologous dendritic cells against CIR-A2 cells (–○–), CIR-A2 pulsed with 25 µg $ml^{-1}$ of PTH-rP (PTR-4) peptide (–●–) CIR-A2 cells transfected with pc3-DNA (–●–), CIR-A2 cells transfected with PTH-rP plasmid (GC90) (–○–), HLA-A2.1⁺/PTH-rP⁻ colon carcinoma SW1463 cells (–□–), HLA-A2.1⁺ /PTH-rP⁺ prostate carcinoma LNCaP cells (–■–), LNCaP cells in presence of anti-HLA-A2.1 mAb (dilution 1:100) (–■–), LNCaP cells in presence of a control isotype mAb (UPC-10) (–□–), PTH-rP⁺ autologous metastatic prostate cancer M-CaP cells in presence of UPC-10 (–▲–), and M-CaP cells in presence of an anti-HLA-A2.1 (A2,69) mAb (–△– ). Data shown in the figure are derived from 3 different experiments for each target cell line with similar results. In the cytotoxic tests the differences between the values from PTH-rP peptide pulsed- and PTH-rP transfected-CIR-A2 compared with values from unpulsed CIR-A2 and pcDNA3 trasfected CIR-A2 cells, respectively were found statistically significant ($P < 0.05$); the values from LNCaP cells in presence of A2.69 and SW-1463 cells with the values from LNCaP cells in presence of UPC-10 and LNCaP cells, respectively were found statistically significant ($P < 0.05$); the values from M-CaP cells in presence of UPC-10 and M-CaP cells in presence of A2.69 respectively, were found statistically significant ($P < 0.05$)

### HLA-A2.1 restriction of prostate cancer CTL-mediated cytolysis

TM-PTR-4-mediated target cell cytolysis was restricted by HLA-A2.1 since tumour cell killing was abrogated by the anti-class I mAb (A2.69) (Figure 1), however prostate cancer cell cytolysis by the TM-PTR-4 T cell line was not affected by the treatment with a A2.69 isotype control antibody not reacting with target cells (Figure 1).

### Bone metastases-derived TIL do not have any cytotoxic activity against target cells

Progenitor TIL not stimulated with PTH-rP epitope peptide, were also investigated for their anti-tumour activity in 6- and 18-hour cytotoxic assays. The TIL were maintained in culture for 6 and 8 weeks in presence of IL-2, and stimulated every 15 days with autologous irradiated prostate cancer cells and autologous DC. This T cell culture (TM-TIL), was not able to lyse LNCaP, SW-1463, unpulsed or PTR-4 peptide pulsed CIR-A2 targets, as well as autologous prostate cancer cells (Figure 3). Cytotoxic activity of patient TIL, in vitro stimulated with peptide-pulsed DC derived from PBMC isolated prior patient cytokine therapy, was tested against the same target cells after 4, 6 and 8 in vitro stimulations. In none of these experiments a PTH-rP as well as a PTH-rP peptide-specific cytotoxic activity could be demonstrated (data not shown). The same results were also obtained when peptide-pulsed autologous (pre or post treatment) DC were used to stimulate in vitro patient PBMC instead of TIL (data not shown).

*© 2001 Cancer Research Campaign*

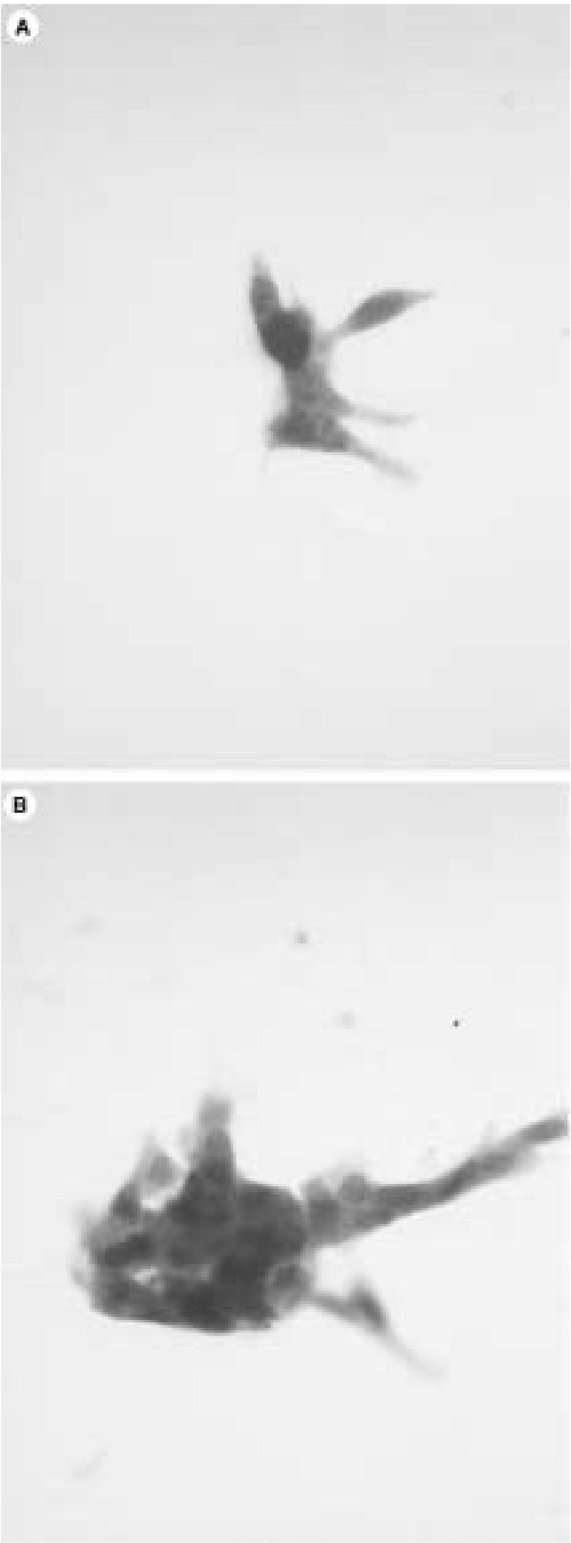

**Figure 2** Expression of prostate-specific antigen (PSA) in metastatic prostate carcinoma M-CaP cells evaluated by immunohistochemistry with labelled monoclonal antibodies. Intense cytoplasmic prostate-specific antigen (PSA) expression is evident in both metastatic prostate carcinoma M-CaP cells (**A**) and prostate carcinoma cell line (LNCaP) used a positive control (**B**). (Avidin–Streptavidin method; 375× original magnification)

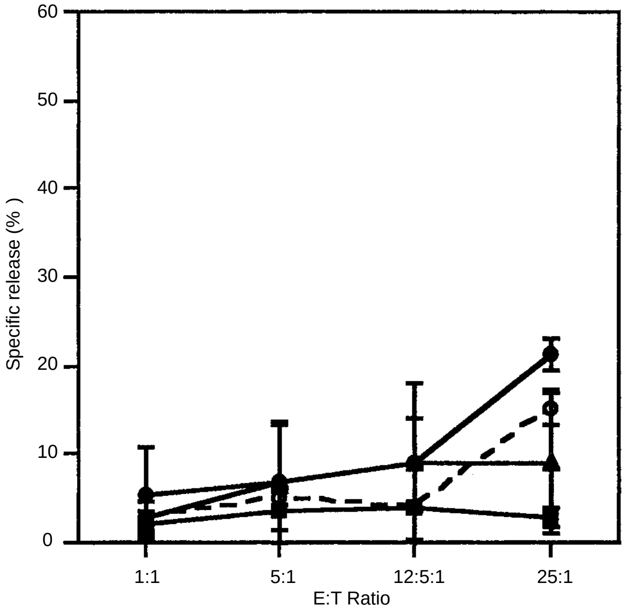

**Figure 3** 6 hour $^{51}Cr$ release cytotoxic assay. Cytotoxic activity of the human (TM-TIL), TIL line against CIR-A2 cells (—○—), CIR-A2 pulsed with 25 µg ml$^{-1}$ of PTH-rP (PTR-4) peptide (—●—), HLA-A2.1$^{+}$/PTH-rP$^{+}$ prostate carcinoma LNCaP cells (—■—), PTH-rP$^{+}$ autologous metastatic prostate cancer M-CaP cells (—▲—). Data shown in the figure are derived from 3 different experiments for each target cell line with similar results. In the cytotoxic tests the differences between the values were never found statistically significant ($P > 0.05$)

### PTH-rP peptide-specific cytotoxic activity of a CTL line generated from a normal donor

The PTH-rP and PTH-rP peptide-specific cytotoxic activity of the CTL line derived from the normal donor was also investigated. In 18-hour cytotoxic assays it was observed that the latter CTL line, designated normal donor (ND)-PTR-4, was able to kill PTR-4 pulsed or PTH-rP gene transfected CIR-A2 target cells and exerted a minimal HLA-A2.1 restricted cytotoxic activity against LNCaP cells (Figure 4). These T lymphocytes were conversely, unable to kill: the peptide unpulsed CIR-A2 cells; the same targets cells transfected with the plasmid vector (pcDNA3), or pulsed with the negative control peptide (CEA-derived) with HLA-A2.1 binding motifs; and the colon carcinoma, SW-1463 cells (Figure 4).

In contrast with TM-PTR-4, the PTR-4 specific CTL line (ND-PTR-4) derived from the normal donor was unable to kill the M-CaP cells, did not exert any cytotoxic activity in 6 hour $^{51}Cr$ release assays (data not shown in figure) and exerted a lower level of cytotoxic activity against PTR-4 pulsed and GC90 transfected CIR-A2 and LNCaP target cells (Figures 1 and 4).

### DISCUSSION

The presence of TIL has been considered an expression of a host immune reaction against the cancer cells (Vose and Moore, 1985; Whiteside, 1991). Once activated and expanded in vitro they have been found to have an effective antitumour activity against autologous tumour cells (Vose and Moore, 1985; Topalian et al, 1987; Whiteside, 1991). On the basis of these findings, several clinical trials have been designed in order to reinfuse autologous ex vivo expanded TIL (together with IL-2 administration) for the treatment of immunogenic cancers such as malignant melanoma and

© 2001 Cancer Research Campaign

**Table 2** HLA-A2.1 molecule expression and PTH-rP production in lymphocyte target cells

| Target cells | HLA-A2.1 expression (%)* | PTH-rP production (pg $ml^{-1} \times 10^6$ cells)# |
|---|---|---|
| CIR-A2 | 98.5 (2.2) | Not detectable |
| CIR-A2 transfected with pcDNA3 | 97.2 (4.24) | Not detectable |
| CIR-A2 transfected with GC90 (PTH-rP gene plasmid) | 96.6 (3.23) | 13.56 (5.6) |
| LNCaP | 29.8 (3.7) | 15.2 (5.5) |
| M-CaP | 60.1 (2.2) | 25.1 (3.5) |
| SW1463 | 75 (3.2) | Not detectable |

*HLA-A2.1 expression was evaluated by indirect immunofluorescence using an anti-HLA-A2.1 mAb (A2.69) and a FICT conjugated goat-anti-mouse. Results are expressed as percentage of fluorescent cells. Marker expression was considered negative when lower than 4%. Results are expressed as percentage of each cell sample reactive with mAb. Routinely 2–4% of cells are stained when treated either with no priming mAb or an isotype related control mAb. #PTH-rP was evaluated by a sandwich immunoradiometric assay (IRMA); values lower than 1.5 pg were considered negative. *#Numbers in parentheses represent standard deviations (SD).

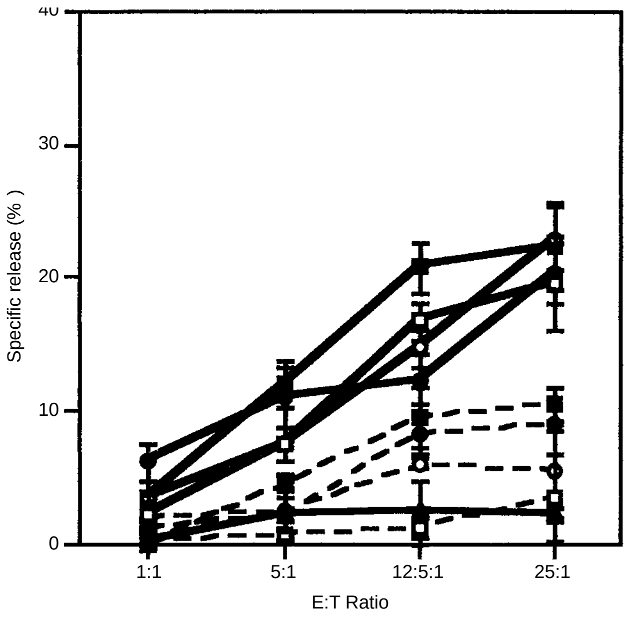

**Figure 4** 18 hour $^{51}Cr$ release cytotoxic assay. Cytotoxic activity of human PBMC stimulated in vitro with IL-2 and PTH-rP peptide-pulsed autologous dendritic cells against CIR-A2 cells (—○—), CIR-A2 pulsed with 25 μg $ml^{-1}$ of PTH-rP (PTR-4) peptide (—●—), CIR-A2 cells transfected with pc3-DNA (—●—), CIR-A2 cells transfected with PTH-rP plasmid (GC90) (—○—) HLA-A2.1$^+$/PTH-rP$^-$ colon carcinoma SW1463 cells (—□—), HLA-A2.1$^+$/PTH-rP$^+$ prostate carcinoma LNCaP cells (—■—), LNCaP cells in presence of anti-HLA-A2.1 mAb (dilution 1:100) (—■—), LNCaP cells in presence of a control isotype mAb (UPC-10) (—□—), PTH-rP$^+$ autologous metastatic prostate cancer M-CaP cells (—▲—). Data shown in the figure are derived from 3 different experiments for each target cell line with similar results. In the cytotoxic tests the differences between: the values from PTH-rP peptide-pulsed-and PTH-rP transfected-CIR-A2 compared with values from unpulsed CIR-A2 and pcDNA3 trasfected CIR-A2 cells, respectively were found statistically significant ($P < 0.05$); the values from LNCaP cells in presence of A2.69 and SW-1463 cells with the values from LNCaP cells in presence of UPC-10 and LNCaP cells, respectively were found statistically significant ($P < 0.05$); the values from M-CaP cells with the values from SW-1463 and CIR-A2 cells were found statistically not significant ($P > 0.05$)

renal cell carcinoma (Topalian et al, 1987; Whiteside, 1991; Arienti et al, 1993).

The results of these trials have shown that this therapeutic approach is well tolerated and has a low level of toxicity, which is mainly produced by IL-2 administration, but the antitumour efficacy of the protocols is still debated because the studies have been sporadic and generally based on small patient populations (Arienti et al, 1993). The role of TIL in the primitive or metastatic neoplasia in vivo has not yet been defined but, although tumour antigen-specific cytotoxic activity has been demonstrated in vitro (Topalian et al, 1987; Arienti et al, 1993; Whiteside and Parmiani, 1994; Pierce et al, 1995), there is clear evidence that for some reason they are unable to eliminate cancer cells from the site where they were extracted.

The results of our study provides demonstration that an antigen derived CTL epitope (PTR-4) peptide pulsed DC can be used to generate in vitro a PTH-rP peptide CTL line from lymphocytes infiltrating a prostate carcinoma bone metastatic lesion. We have recently, characterized several human CTL lines specific for PTR-4 and other PTH-rP peptides, generated by in vitro stimulation of HLA-A2.1$^+$ healthy donor PBMC with low dose IL-2 and peptide-pulsed autologous DC. These CTL lines were able to kill in HLA-2.1 restricted manner, CIR-A2 targets pulsed with the specific peptide used for the CTL line stimulation and HLA-2.1$^+$ prostate (LNCaP) and breast carcinoma cells (MDA-MB-231) producing PTH-rP. Cold competition assays also demonstrated the peptide specificity of these CTL lines since their cytotoxic activity against both LNCaP and MDA-MB-231 target cells was abrogated by the addition in the cytotoxic assays of cold competitors (T2-A2 target cells) previously pulsed with the specific PTH-rP epitope peptide (Francini et al, 2001).

In the present study we found that the PTR-4-specific CTL line generated from the prostate carcinoma patient TIL had similar immunological features as those observed in the CTL lines derived from the healthy donor. While the PTH-rP and PTHrP peptide specificity of these CTL lines was very close, significant differences were observed in regard to the amount of cytotoxicity and to the time necessary to kill the target cells. TM-PTR-4 cells were, in fact, able to lyse the specific target cells in 4–6 hour cytotoxic assays while the CTL derived from normal donor needed at least 18 hours. In addition, the CTL derived from the normal donor were unable to kill in vitro the metastatic prostate cancer cells of the patient (M-CaP). The lack in cytolytic activity of ND-PTR-4 CTL line against M-CaP might be explained considering that the healthy donor lymphocytes and the tumour cells are not autologous even though they match for HLA-A2.1 expression; nevertheless, it is also possible that the cytotoxic activity of this CTL line is not strong enough to kill the target cells rapidly. The longer

*© 2001 Cancer Research Campaign*

interval of time needed to kill the target cells might give the chance to the tumour cells to activate their own mechanisms of defence and eventually to fight back and kill the lymphocytes. At this purpose several in vitro studies on various tumour cell lines clearly demonstrated that tumour cells possess different mechanisms of resistance to various cytotoxic immune-effectors (natural killer (NK), lymphokine activated killer (LAK), TIL, etc) and that such mechanisms may be modulated in different way with several drugs or biological response modifiers (Correale et al, 1991, 1992, 1995; Frost et al, 1997; Tagliaferri et al, 1998). To provide a stronger explanation for this finding further experiments are presently, in progress to investigate whether ND-PTR-4 (derived from the normal donor PBMC) and TM-PTR-4 (derived from the patient TIL) CTL lines utilize the same molecular mechanism to induce target cell killing (FAS ligand, TNFα, perphorines etc.).

In this study patient PBMC, in vitro stimulated with peptide-pulsed autologous DC as well as progenitor TIL, in vitro stimulated with autologous tumour cells and DC, were not able to kill any of the same targets including the autologous cancer cells. Although these findings need to be confirmed on a larger number of patients, we can still hypothesize that we were unable to establish a PTH-rP-specific CTL line from the PBMC because during the natural history of the disease, the peripheral blood of this patient was depleted of PTH-rP peptide-specific precursors which were gathered and inactivated in the metastatic site where PTH-rP producing cancer cells are localized. The in vitro stimulation of TIL with epitope peptides pulsed DC may stimulate such precursors present in high frequency among the lymphocytes infiltrating the metastatic lesion and eventually rescue their antigen-specific antitumour activity, which does not seem irremediably altered by tumour cells as reported in literature (Melero et al, 1997). Peptide-pulsed DC were chosen to stimulate the TIL in order to induce an efficient PTH-rP-specific CTL response because of their powerful antigen-presenting ability. There is evidence indicating that DC are the most powerful known APC (Grabbe et al, 1995; Bell et al, 1999), and it known that they play a critical role in antigen presentation in vivo and in the induction of antigen-specific CTL responses. Although tumour cells express antigen peptides recognized by CTL, most tumours lack the co-stimulatory molecules necessary for CTL activation (Melero et al, 1997). Antigen-specific CTL activation and clonal expansion needs in fact, co-stimulatory surface molecule interactions between effector-precursors (CD28) and antigen peptide-presenting cells (CD80/B7.1; CD86/B7.2), such as DC, activated B lymphocytes, and monocytes (Grabbe et al, 1995; Melero et al, 1997; Bell et al, 1999). Unlike other member of the APC family, DC are capable of inducing primary immune responses by activating naive T cells (Markowicz and Engleman, 1990; Grabbe et al, 1995; Bell et al, 1999; Liuykx-de Bakker et al, 1999). The DC used in this study were derived from a prostate cancer patient enrolled in a clinical trial after he had received GM-CSF and IL-2. The biological results of that study, which tested the effects of a sequential combination of the 2 cytokine, showed that the treatment affects the immunocompetence of cancer patients (Correale et al, 2001), a finding that may have a direct clinical application as the cell context in which an antigen peptide is presented to the immune system is critical for the induction of an efficient antigen-specific immune response (Panelli and Marincola, 1998; Rosenberg et al, 1998). The study showed that the administration of human recombinant (hr) GM-CSF and hr IL-2 increases the percentage of PBMC-expressing bone marrow-derived DC markers and also promotes DC maturation and functions, as suggested by the finding of a higher expression of CD83, CD80 and CD86 on PBMC populations gated as DC (Correale et al, 2001).

In order to estimate the DC enrichment, patient PBMC and the derivative cultures were examined for the expression of CD1a, HLA-I, HLA-DR, CD11c, CD86, CD83, and CD80, all which are known to be markers highly expressed by activated DC (Bell et al, 1999) and to be involved in T cell co-stimulation and activation.

This study also demonstrated that the DC derived from a patient with prostate cancer who had received GM-CSF and IL-2 are able to stimulate tumour infiltrating initiating and can induce an antigen-specific immune reaction with antitumour activity. PTH-rP peptide-pulsed DC derived from pretreatment patient PBMC were not able to generate any PTH-rP-specific CTL response in autologous TIL. This result suggests that the cytokine treatment induced functional modification in circulating DC that results in an increased antigen-presenting ability. This hypothesis is supported by the recent finding that GM-CSF and IL-2 administered in cancer patients enhance the antigen-presenting ability of their PBMC increasing their proliferative response to influenza antigens (Correale et al, 2001).

The results of the present study could have an immediate clinical application as PTH-rP is over-expressed in 90% of primary prostate carcinoma and 90% of epithelial malignancies derived from bone metastases (Guise, 1997). A number of authors have reported that PTH-rP mediates the interaction of tumour cells with normal osteoclast cells, which ultimately promote tumour cell survival and growth in the bone, and the same authors consider PTH-rP a molecular structure that is indispensable for the formation of bone metastases from prostate, breast and lung carcinoma (Guise, 1997). Recognition by cytotoxic T-cell lines suggests that PTR-4 peptide-based vaccine therapy and PTR-4-specific TIL adoptive reinfusions may be potential candidates for use in the treatment of prostate carcinoma bone metastases.

### ACKNOWLEDGEMENTS

The authors wish to thank the paramedic personnel of the Medical Oncology Division, University of Siena, for their critical help for sample assistance, management, and care. This study was supported by grants from the Italian Ministry of University, Research and Technological Development (MURST, Progetto inter-universitario ex-40%, 1998–2000) and from the Italian National Research Council (CNR) (Italy–USA exchange program).

### REFERENCES

Abrams SI, Dobrzanski MJ, Wells DT, Stanziale SF, Zaremba S, Masuelli L, Kantor JA and Sclom J (1995) Peptide-specific activation of cytotoxic CD4+ T lymphocytes against tumor cells bearing mutated epitopes of K-ras p21. *Eur J Immunol* **25**: 2588–2597

Arienti F, Belli F, Rivoltini L, Gambacorti-Passerini C, Furlan L, Mascheroni L, Prada A, Rizzi M, Marchesi E, Vaglini M, Parmiani G and Cascinelli N (1993) Adoptive Immunotherapy of advanced melanoma patients with interleukin-2 (IL-2) and tumor infiltrating lymphocytes selected in vitro with low doses of IL- 2. *Cancer Immunol Immunother* **36**: 315–322

Bell D, Young JW and Banchereau J (1999) Dendritic cells. *Adv Immunol* **72**: 255–324

Boyum A (1968) A one stage procedure for isolation of granulocytes and lymphocytes from human blood. General sedimentation properties of white blood cells in a 1g gravity field. *Scand J Clin Lab Invest* **97**(**Suppl**): 51–76

Cerundolo V, Alexander J, Anderson K, Lamb C, Cresswell P, McMichael A, Gotch F and Townsend A (1990) Presentation of viral antigen

© 2001 Cancer Research Campaign controlled by a gene in the major histocompatibility complex. *Nature* **345**: 449–452

Coffey DS (1993) Prostate Cancer. An overview of an increasing dilemma. *Cancer* **71**: 880–886

Correale P, Tagliaferri P, Celio L, Genua G, Montagnani S and Bianco AR (1991) Verapamil upregulates sensitivity of human colon and breast cancer cells to LAK-cytotoxicity in vitro. *Eur J Cancer* **27**: 1393–1395

Correale P, Procopio A, Celio L, Caraglia M, Genua G, Coppola V, Pepe S, Normanno N, Vecchio I, Palmieri G, Montagnani S, Tagliaferri P and Bianco AR (1992) Phorbol Myristate Acetate induces resistance of human melanoma cells to natural killer- and lymphokine activated killer (LAK) -mediated cytotoxicity. *Cancer Immunol Immunother* **34**: 272–278

Correale P, Caraglia M, Fabrocini A, Guarrasi R, Pepe S, Patella V, Marone G, Pinto A, Bianco AR and Tagliaferri P (1995) Bryostatin 1 enhances lymphokine activated killer (LAK) sensitivity and modulates very late activation antigen (VLA) profile of cultured human tumor cells. *Anticancer Drugs* **6**: 285–290

Correale P, Walmsley K, Nieroda C, Zaremba S, Zhu M, Schlom J and Tsang KY (1997) *In vitro* generation of human cytotoxic T lymphocytes specific for peptides derived from prostate-specific antigen. *J Natl Cancer Inst* **89**: 293–300

Correale P, Campoccia G, Tsang KY, Micheli L, Cusi MG, Sabatino M, Bruni G, Sestini S, Petrioli R, Pozzessere D, Marsili S, Fanetti G, Giorgi G and Francini G (2001a) Recruitment of dendritic cells and enhanced antigen specific immune-reactivity in cancer patients treated with hrGM-CSF (molgramostim) and hr IL-2: results from a Phase Ib Clinical Trial. *Eur J Cancer* **37**(7): 892–902

Correale P, Cusi MG, Sabatino M, Micheli L, Pozzessere D, Nencini C, Valensin PE, Giorgi G, Zurbriggen R, Gluck R and Francini G (2001b) Tumour associated antigen (TAA) specific cytotoxic T cell (CTL) response in vitro and in a mouse model, induced by TAA-plasmids delivered by influenza virosomes. *Eur J Cancer* (in press)

Fenton RG, Taub DD, Kwak LW, Smith MR and Longo DL (1993) Cytotoxic T-cell response and in vivo protection against tumor cells harboring activated ras proto-oncogenes. *J Natl Cancer Inst* **85**: 1294–1302

Francini G, Petrioli R, Manganelli A, Cintorino M, Marsili S, Aquino A and Mondillo S (1993) Weekly chemotherapy in advanced prostatic cancer. *Br J Cancer* **67**(6): 1430–1436

Francini G, Pozzessere D, Campoccia G, Sabatino M, Petrioli R, Fanetti G, Lozzi L, Neri P and Correale P (2001) Generation and characterization of Human Cytotoxic T Lymphocytes Specific For HLA-A2-1 Binding Peptides Derived From Parathyroid-related Protein. *Submitted for publication*.

Frost P, Ng CP, Belldegrun A and Bonavida B (1997) Immunosensitization of prostate carcinoma cell lines for lymphocytes (CTL, TIL, LAK) mediated apoptosis via the Fas-Fas-ligand pathway of cytotoxicity. *Cell Immunol* **180**(1): 70–83

Grabbe S, Beissert S, Schwarz T and Granstein RD (1995) Dendritic cells as initiators of tumor immune responses: a possible strategy for tumor immunotherapy? *Immunol Today* **16**: 117–121

Guadagni F, Witt PL, Robbins PF, Schlom J and Greiner WJ (1990) Regulation of carcinoembryonic antigen expression in different human colorectal tumor cells by interferon-$\alpha$. *Cancer Res* **50**: 6248–6255

Guise TA (1997) Parathyroid hormone–related protein and bone metastases. *Cancer* **80**: 1572–1580

Hortobagyi G (1991) Bone metastases in breast cancer patients. *Semin Oncol* **18**(5): 11–15

Houbiers JG, Nijman HW, van der Burg SH, Drijfhout JW, Kenemens P, van de Velde CJ, Brand M, Momberg F, Kast WM and Melief CJM (1993) In vitro induction of human cytotoxic T lymphocyte responses against peptides of mutant and wild-type p53. *Eur J Immunol* **23**: 2072–2077

Jung S and Schluesener HJ (1991) Human T lymphocytes recognize a peptide of single point-mutated oncogene ras proteins. *J Exp Med* **173**: 273–276

Liuykx-de Bakker SA, Gruijl TD, Scheper RJ, Wagstaff J and Pinedo HM (1999) Dendritic cells: a novel therapeutic modality. *Ann Oncol* **10**: 21–27

Marincola FM (1994) Interleukin 2. *Biol Ther Cancer Updates* **4**: 1–16

Markowicz S and Engleman EG (1990) Granulocyte-macrophage colony stimulating factor promotes differentiation and survival of human peripheral blood dendritic cells in vitro. *J Clin Invest* **85**: 955–961

Melero I, Bach N and Chen L (1997) Costimulation, tolerance and ignorance of cytolytic T lymphocytes in immune responses to tumor antigens. *Life Sciences* **60**: 2035–2041

Panelli MC and Marincola F (1998) Immunotherapy update: from interleukin-2 to antigen specifictherapy. *Educational Book from the 34th Annual Meeting of the American Society of Clinical Oncology*. pp 467–469

Pierce WC, Belldegrun A and Figlin RA (1995) Cellular therapy: scientific rationale and clinical results in the treatment of metastatic renal-cell carcinoma. *Semin Oncol* **22**(1): 74–80

Restifo NP and Sznol M (1997) Cancer Principles and Practice of Oncology. Lippincott: Philadelphia

Romani N, Reider D, Heuer M, Ebner S, Kämpgen E, Eibl B, Niederwieser D and Schuker G (1996) Generation of mature dentritic cells from human blood. An improvement method with special reagent to clinical applicability. *J Immunol Methods* **196**: 137–151

Rosenberg SA, Yang J, Schwartzentruber DJ, Rosenberg SA, Yang JC, Schwartzentruber DJ, Hwu P, Marincola FM, Topalian SL, Restifo NP, Sznol M, Schwarz SL, Spiess PJ, Wunderlich JR, Seipp CA, Einhorn JH, Rogers Freezer L and White DE (1998) Impact of cytokine administration on the generation of antitumor reactivity in patients with metastatic melanoma receiving a peptide vaccine. *J Immunol* **163**: 1690–1695

Rosenberg SA, Yannelli JR, Yang JC, Topalian L, Schwartzentruber J, Weber S, Parkinson R, Seipp A, Einhorn HD and White E (1994) Treatment of patients with metastatic tumor infiltrating lymphocytes and interleukin 2. *J Natl Cancer Inst* **86**: 1159–1166

Rubens RD (1998) Bone metastases – the clinical problem. *Eur J Cancer* **34**: 210–213

Tjoa BA, Simmons SJ, Bowes VA, Ragde H, Rogers M, Elgamal A, Kenny GM, Cobb OE, Ireton RC, Troychak MJ, Salgaller ML, Boynton AL and Murphy GP (1998) Evaluation of Phase I/II clinical trial in prostate cancer with dendritic cells and PSMA peptides. *Prostate* **36**: 39–44

Tagliaferri P, Guarrasi R, Caraglia M, Morelli D, Fabbrocini A, Correale P and Bianco AR (1998) Tumour cell resistance to non MHC-restricted lymphocytes: molecular mechanisms and clinical implications. *Cancer Immunol Immunother* **46**: 121–127

Topalian SL and Rosenberg SA (1989) Tumour specific cytolysis by lymphocytes infiltrating human melanomas. *J Immunol* **142**: 3714–3725

Topalian SL, Muul LM, Solomon D and Rosenberg SA (1987) Expansion of human tumor infiltrating lymphocytes for use in immunotherapy trials. *J Immunol Methods* **102**: 127–131

Tsang KY, Nieroda CA, De Filippi R, Chung YK, Yamaue H, Greiner JW and Schlom J (1994) Induction of human cytotoxic T- cell lines directed against point-mutated p21 ras-derived synthetic peptides. *Vaccine Res* **3**: 183–193

Tsang KY, Zaremba S, Nieroda CA, Zhu MZ, Hamilton JM and Schlom J (1995) Generation of human cytotoxic T cells specific for human carcinoembrionic antigen epitopes from patients immunized with recombinant vaccinia CEA vaccine. *J Natl Cancer Inst* **87**: 982–990

Van der Bruggen P, Traversari C, Chomez P, Lurquin C, De Plaen E, Van den Eynde B, Knuth A and Boon T (1991) A gene encoding an antigen recognized by cytotoxic T lymphocytes on a human melanoma. *Science* **254**: 1643–1647

Vinholes J, Coleman R and Eastell R (1996) Effects of bone metastases on bone metabolism: Implications diagnosis, imaging and assessment of response to cancer treatment. *Cancer Treatment Reviews* **22**: 289–331

Vose BM and Moore M (1985) Human tumor infiltrating lymphocytes: a marker of host response. *Semin Hematol* **22**: 27–29

Whiteside TL (1991) Tumour-infiltrating lymphocytes as antitumor effector cell. *Biotherapy* **5**: 47–61

Whiteside TL and Parmiani G (1994) Tumour infiltrating lymphocytes: their phenotype, functions and clinical use. *Cancer Immunol Immunother* **39**: 15–21

 *© 2001 Cancer Research Campaign*
